# The Fanconi Anemia Core Complex Is Dispensable during Somatic Hypermutation and Class Switch Recombination

**DOI:** 10.1371/journal.pone.0015236

**Published:** 2010-12-29

**Authors:** Peter H. L. Krijger, Niek Wit, Paul C. M. van den Berk, Heinz Jacobs

**Affiliations:** Division of Immunology, The Netherlands Cancer Institute, Amsterdam, The Netherlands; University of Minnesota, United States of America

## Abstract

To generate high affinity antibodies during an immune response, B cells undergo somatic hypermutation (SHM) of their immunoglobulin genes. Error-prone translesion synthesis (TLS) DNA polymerases have been reported to be responsible for all mutations at template A/T and at least a fraction of G/C transversions. In contrast to A/T mutations which depend on PCNA ubiquitination, it remains unclear how G/C transversions are regulated during SHM. Several lines of evidence indicate a mechanistic link between the Fanconi Anemia (FA) pathway and TLS. To investigate the contribution of the FA pathway in SHM we analyzed FancG-deficient B cells. B cells deficient for FancG, an essential member of the FA core complex, were hypersensitive to treatment with cross-linking agents. However, the frequencies and nucleotide exchange spectra of SHM remained comparable between wild-type and FancG-deficient B cells. These data indicate that the FA pathway is not involved in regulating the outcome of SHM in mammals. In addition, the FA pathway appears dispensable for class switch recombination.

## Introduction

Within the germinal center (GC), antigen activated B cells undergo class switch recombination (CSR) and somatic hypermutation (SHM). During CSR the immunoglobulin (Ig) heavy chain constant region is replaced for a downstream constant region, to generate an antibody with a different effector function. CSR depends on the introduction of double strand breaks in two active switch regions of the Ig heavy chain constant regions and involves nonhomologous end-joining (NHEJ) to ligate the break sites. [1]. To generate high affinity antibody variants, GC B cells can introduce point mutations into the variable region of their rearranged immunoglobulin (Ig) genes. This process of SHM occurs at an extraordinary rate of one in a thousand base pairs per generation [2]. To model the underlying mechanism, error-prone polymerases were postulated about half a century ago [3]. Yet, only the last two decades revealed the existence of such DNA polymerases. In contrast to replicative DNA polymerases, TLS polymerases are highly mutagenic when replicating across undamaged DNA [4], [5]. At least polymerase η, Rev1 and to some degree polymerase κ have been related to SHM. Since each polymerase displays its own mutation signature, alterations in the nucleotide exchange spectrum can often be attributed retrospectively to the absence of, or failure in activating specific polymerases. For example, Rev1-deficient B cells display a selective reduction of G/C to C/G transversions [6]–[8], a finding consistent with the restricted dCMP transferase activity of Rev1 [9]. In contrast, the mutation spectra of polymerase η -deficient B cells from human and mice lack a significant fraction of A/T mutations [10]–[12]. While the lack of polymerase κ had no effect on SHM [13], polymerase κ was found to generate A/T mutations in the absence of polymerase η [14]. Recently, it has been demonstrated that SHM at template A/T is regulated by site specific monoubiquitination of proliferating cell nuclear antigen (PCNA) at lysine 164 (PCNA-Ub). In agreement with an important role for PCNA-Ub in recruiting and activating TLS polymerases upon replication fork stalling [15]–[17], analysis of the mutation spectra of mutated Ig genes in B cells from PCNA^K164R^ knock-in mice revealed a selective 10-fold reduction of A/T mutations [18], [19]. Consistently, PCNA knock-out mice reconstituted with a PCNA^K164R^ transgene showed a reduction of A/T mutations in Ig genes [20], suggesting that during SHM PCNA-Ub recruits polymerase η and κ to introduce mutations at template A/T. The question remains, what are the molecular prerequisites that stimulate error-prone polymerases like Rev1 to establish transversions at template G/C?

Fanconi anemia (FA) is an autosomal recessive genetic disorder, which at the cellular level is characterized by a hypersensitivity to DNA cross-linking agents such as Cisplatin [21]. How the FA pathway mediates resistance to cross-links is largely unknown. Current models suggest that after replicative DNA polymerases are stalled at a DNA cross-link, FANCD2 and FANCI become monoubiquitinated by the FA core complex. The FA core complex consists of eight essential FA proteins, FANCA, -B, -C, -E, -F, -G, -L, -M, and two FA-Associated Proteins FAAP100 and FAAP24. FANCD2 was shown to stimulate incision of one of the strands containing the cross-link and to recruit TLS polymerases to enable a direct replicative bypass [22]. In agreement, the TLS polymerases Rev1 and Rev3 have been demonstrated to act synergistically with the FA pathway for cross-link repair in chicken DT40 B cells [23]. In addition, it has been reported recently, that FANCD2 modifies the resulting double strand break to prevent Ku70 from binding and activating NHEJ [24], [25].

As the FA pathway has been associated with damage tolerant TLS polymerases, including Rev1, we questioned whether this pathway controls the outcome of SHM in mammals. Interestingly, it has been reported that chicken DT40 B cells deficient for members of the FA pathway show a decrease in SHM [23], [26], although the precise mechanism for the decrease in the accumulation of non-templated mutations is currently unclear. To determine the role of the FA pathway in SHM, we analyzed SHM in FancG-deficient mice. In addition, given the inhibitory action of FANCD2 on NHEJ we addressed whether the FA pathway is involved in CSR.

## Results and Discussion

### Sensitivity of wild-type and FancG-deficient B cells to cross-linking agents

**Figure 1:**
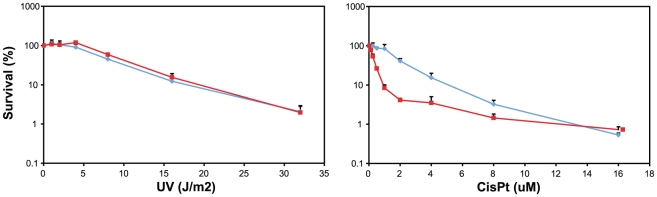
FancG-deficient B cells are sensitive to DNA cross-linking agents. FancG-deficient (red) and wild-type (blue) B cells were stimulated with LPS and exposed to increasing doses of either UV-C (left panel) or Cisplatin (right panel). The percentage of survival after four days of culture is shown.

To investigate the involvement of the FA core complex in SHM we first determined if B cells of FancG-deficient mice display a FA phenotype. Like cells derived from FA patients, mouse embryonic fibroblasts from FancG-deficient mice are unable to monoubiquinate FANCD2 and are highly sensitivity to cross-linking agents such as Cisplatin [27]. To reveal the activity and specificity of the FA pathway in resolving DNA cross-links in primary B cells, B cell cultures from wild-type and FancG-deficient mice were established and exposed to Cisplatin and UV-C. Compared to WT cells FancG-deficient B cells were highly sensitive to Cisplatin, but not to UV-C (Fig. 1). These data are in agreement with those of fibroblast cell lines derived from FA patients and FA-deficient mice. Apparently, the FA pathway is active in primary B cells and plays a role in response to DNA cross-links.

### Mutation frequencies in wild-type and FancG-deficient B cells

**Figure 2:**
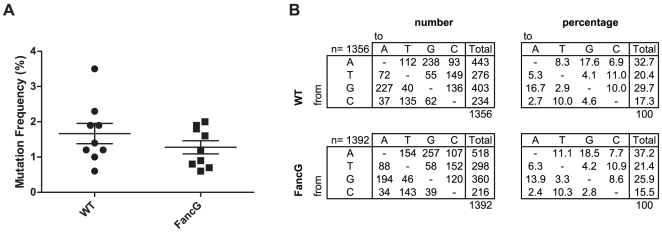
Normal SHM in FancG-deficient B cells. A.) Unaltered accumulation of somatic mutations in germinal center B cells of FancG-deficient mice. The frequency of SHM (% of mutations) as determined from 9 individual mice are shown per genotype (paired Student's t-test, p = 0.3). The mean values and SD are indicated. B.) Normal nucleotide exchange pattern in hypermutated Ig genes of FancG-deficient germinal center B cells. Values are expressed as the total number of mutations (left panel) and percentage of total mutations (right panel). Chi square testing did not reveal any significant changes in the pattern (*p*<0.01).

To determine the contribution of the FA pathway in the regulation of SHM, we sequenced the JH4 intronic region of GC B cells sorted from the Peyer's patches of nine FancG-deficient and nine wild type control mice. By analyzing clonally unrelated introns, 1356 mutations were found in 164 mutated intronic sequences from wild-type B cells and 1392 mutations in 195 mutated intronic sequences from FancG-deficient B cells. In contrast to previous observations made in FA-deficient chicken DT40 B cells, which demonstrated reduced levels of SHM [23], [26], a high frequency of point mutations in somatically mutated Ig genes of both, wild-type and FancG-deficient B cells was found (Table 1). The range of SHM frequencies observed from the individually analyzed mice is depicted in figure 2A. No significant difference in accumulating somatic mutations between wild type and FancG-deficient B cells was found (paired Student's t-test, p = 0.3).

**Table 1:** Mutated JH4 intronic regions from wild-type and FancG-deficient GC B cells.

	WT	FancG −/−
number of mice	9	9
number of mutated sequences	164	195
total number of point mutations	1356	1392
total number of base pairs sequenced	81606	97418
Mutations/base pair (%)	1.7	1.4

### Point mutation spectra in wild-type and FancG-deficient B cells

The decrease in non-templated mutations observed in FA-deficient DT40 cells [23] may relate to impaired TLS activity. Genetic studies have indicated a role for the TLS polymerase Rev1 downstream of the FA pathway upon treatment with cross-linking agents [23]. Furthermore the recruitment of Rev1 upon UV treatment was reported to rely in part on the FA pathway [28]. While DT40 B cells strongly depend on Rev1 for SHM [7], SHM in mammals depends only partly on Rev1 [6], [29]. However, in both systems Rev1-deficient B cells display reduced frequencies of G/C to C/G transversions during SHM [6], [7], [29]. Therefore, if the FA pathway stimulates Rev1 during SHM, a reduction in these mutations is expected in FancG-deficient B cells. To address whether the FA pathway regulates TLS polymerases during SHM in mammals, we analyzed the pattern of non-selected, nucleotide substitutions in the non-transcribed strand of the JH4 intronic region. The spectra of nucleotide substitutions were similar between wild-type and FancG-deficient mice (χ^2^ test, *p*<0.01), indicating that Rev1 and also other TLS polymerases involved in mammalian SHM do not depend on the FA pathway to generate mutations (Fig 2.). The decrease in non-templated mutations found in FA-deficient DT40 cells [23] may relate to an interspecies difference between avian and mammalian SHM. Alternatively, the reduction in mutations observed in FA-deficient DT40 cells is not a consequence of impaired TLS activity, but regulated at a different level. Analysis of the nucleotide exchange pattern in FA-deficient DT40 cells is required to distinguish between these possibilities.

### Class switch recombination in FancG-deficient B cells

**Figure 3:**
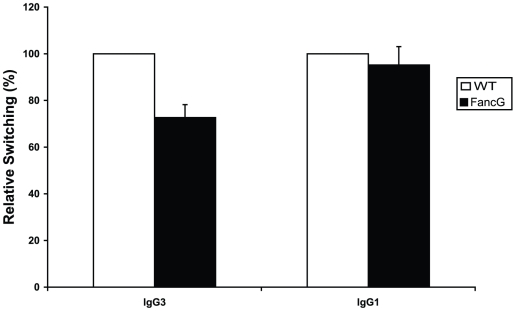
Class switch recombination. Comparison of wild-type (white bars) and FancG-deficient B cells (black bars) switched to IgG3 upon activation by LPS, or switched to IgG1 upon activation by LPS and IL-4. Data represent the mean and SD of individual cultures (n = 3).

Recently, it has been reported that double strand breaks created at cross-links or abasic sites are modified by FANCD2 to prevent Ku70 from binding and activating NHEJ [24], [25]. As CSR depends on NHEJ, we questioned whether the FA pathway regulates class switch recombination by inhibiting NHEJ. To determine the capacity of wild-type and FancG-deficient B cells to undergo CSR to IgG3 and IgG1, naïve B cells were cultured with lipopolysacharide (LPS) in the absence or presence of interleukin-4 (IL-4), respectively (Fig. 3). Inactivation of FancG resulted in a reduction of IgG3 class switched cells compared to wild-type cells. However, as we did not observe a difference in class switching to IgG1, the reduction in IgG3 switching may suggest an isotype-specific role of FA in CSR. Alternatively, the FA pathway does not play a direct role in CSR, but affects the switching process only indirectly. Moreover, as the CSR frequencies did not increase in FancG-deficient B cells, the formal possibility that the FA pathway inhibits CSR by blocking NHEJ can be excluded.

### Concluding remarks

In conclusion, our data indicate for the first time that SHM in mammals is not regulated by the FA pathway. While mutations at template A/T by polymerase η are regulated at the level of PCNA ubiquitination, future studies will have to reveal the underlying molecular mechanism how TLS polymerases like Rev1, involved in the generation of G/C transversions become activated. The normal switching activity to IgG1 and partial reduction to IgG3 suggests, that overall the FA pathway is dispensable for CSR.

## Materials and Methods

### Mice

The generation and genotyping of *FancG*-deficient mice has been described elsewhere [27]. Mice were maintained on pure FVB background at the animal facility of the Netherlands Cancer Institute (Amsterdam, Netherlands). All experiments were approved by an independent animal ethics committee of the Netherlands Cancer Institute (ID 08065 and ID 06003) and executed according to national guidelines.

### Isolation of germinal center B cells and mutation analysis

Germinal center (CD19+, PNA high, CD95+) B cells were sorted from Peyer's patches. Genomic DNA was extracted using proteinase K treatment and ethanol precipitation. The JH4 3′flanking intronic sequence of endogenous rearrangements of VHJ558 family members were amplified during 40 cycles of PCR using PFU Ultra polymerase (Stratagene) [30]. PCR products were purified using the QIAquick Gel Extraction kit (Qiagen) and cloned into the TOPO zero blunt vector (Invitrogen Life Technologies) and sequenced on a 3730 DNA analyzer (Applied Biosystems). Sequence alignment was performed using Seqman software (DNAStar). Calculations exclude non-mutated sequences, insertions and deletions. Clonally related sequences were counted only once.

### Class switch recombination

Naïve splenic B cells from three mice per genotype were obtained by CD43 depletion using biotinylated anti CD43 (Clone S7, BD Biosciences), and the IMag system (BD Biosciences), as described by the manufacturer. Purified B cells were cultured at 10^5^ cells/ml in 24 well plates in IMDM, 8% FCS, 50 µM 2-mercapthoethanol, penicillin/streptomycin and 50 ug/ml E.Coli LPS (055:B5, Sigma) either in the presence or absence of IL-4-containing supernatants generated from X63-m-IL-4 cell cultures. Flow cytometric analysis of surface Ig expression was performed on day 4 of culture using goat anti mouse IgM-APC, IgG1-PE and IgG3-PE (Southern Biotech). Data were analyzed using FlowJo 7.6 software.

### Survival

Naïve splenic B cells were obtained and cultured as described above. For UV-C irradiation, 10^5^ B cells were irradiated in 0.5 ml medium (254 nm, UV Stratalinker® 2400, Stratagene). Following irradiation, cells were cultured in 1 ml complete medium and LPS. For the survival upon Cisplatin induced DNA damage, 10^5^ B cells were grown in 1 ml complete medium and LPS in the continuous presence of different doses of Cisplatin. For determining the survival, B cells were harvested after four days of culture and live (propidium iodine negative) B cells were counted by FACS. Data were analyzed using FlowJo 7.6 software.
